# Supplementary material for: MagIC-Cryo-EM, structural determination on magnetic beads for scarce macromolecules in heterogeneous samples
Source: eLife. 2025 May 20;13:RP103486. doi: 10.7554/eLife.103486 (PMC12092007; doi:10.7554/eLife.103486)
Supplement: Figure 2—figure supplement 1—source data 1. [file elife-103486-fig2-figsupp1-data1.pdf]

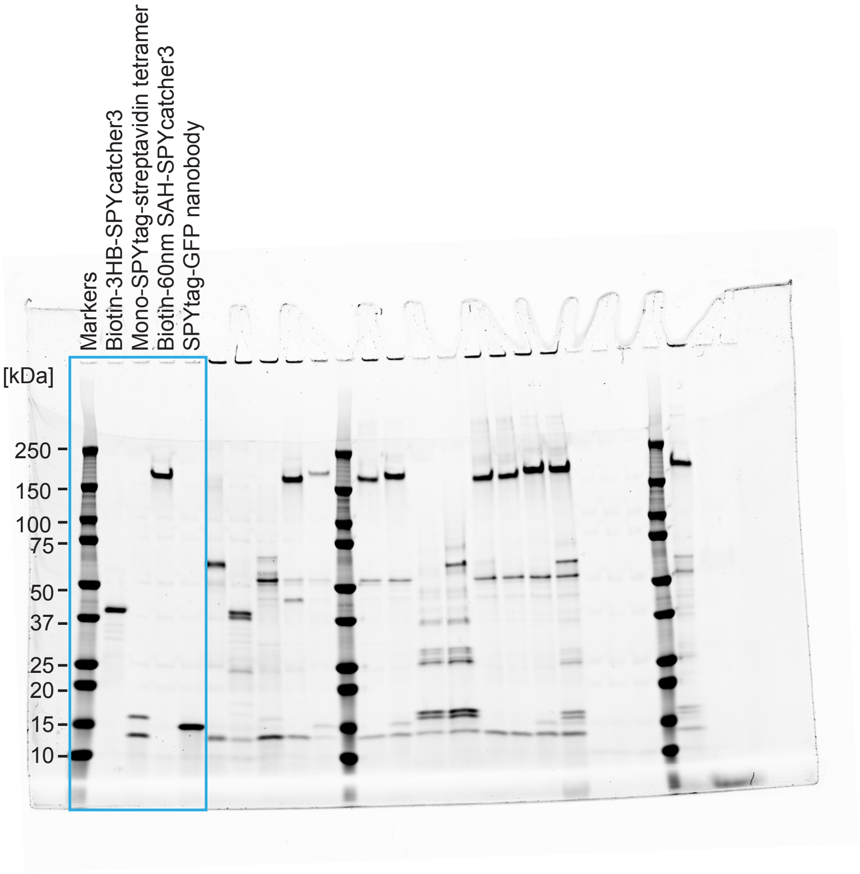

Figure 2—figure supplement 1—source data 1.
Full images of gels and membranes shown in Figure2—figure supplement 1B
