# Supplementary figures and images for: MagIC-Cryo-EM, structural determination on magnetic beads for scarce macromolecules in heterogeneous samples
Source: eLife. 2025 May 20;13:RP103486. doi: 10.7554/eLife.103486 (PMC12092007; doi:10.7554/eLife.103486)

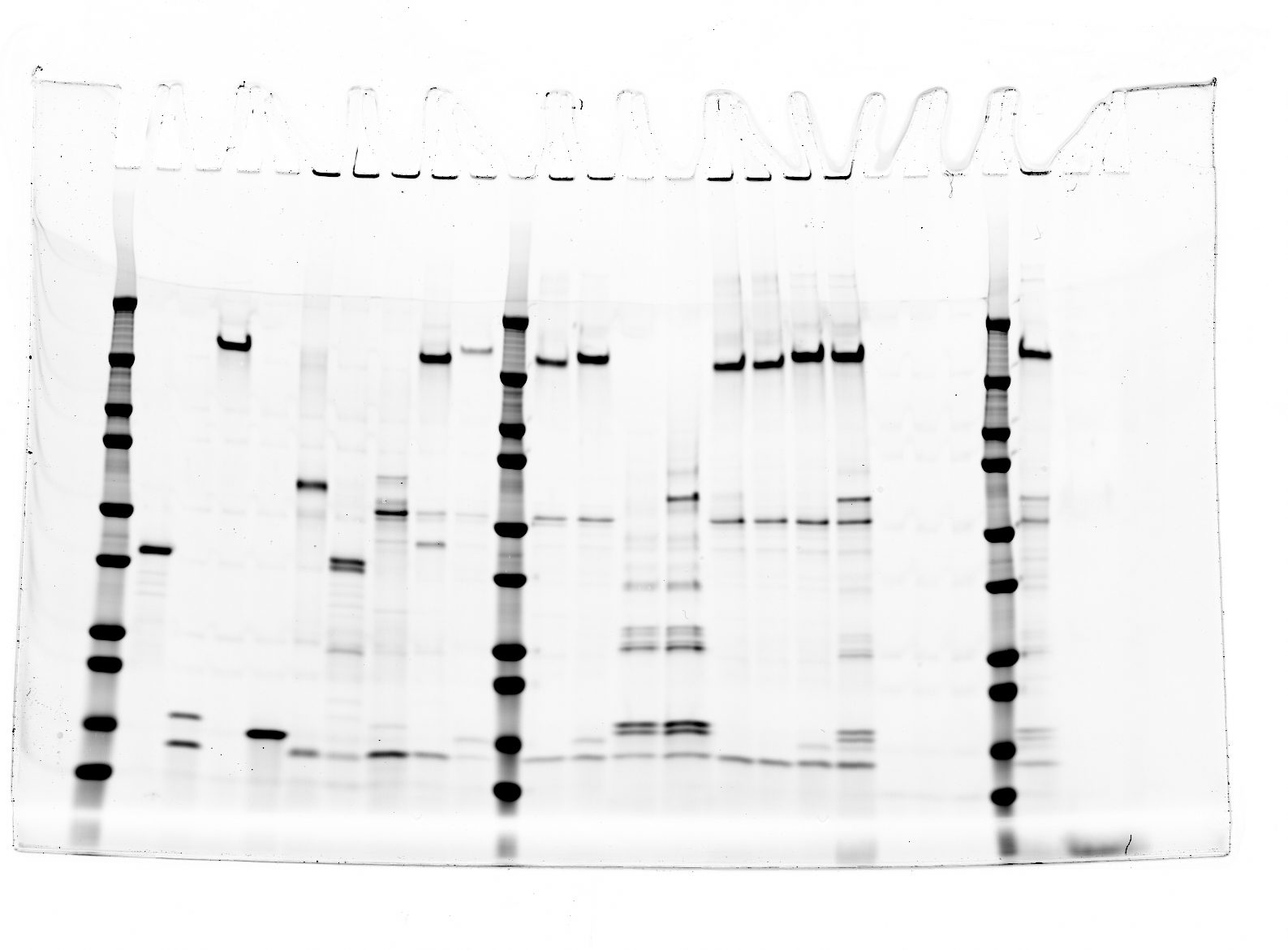

Supplement: Figure 2—figure supplement 1—source data 2. [file elife-103486-fig2-figsupp1-data2.zip › Fig2_Supple1_source_data_2/0001842_01_685Ex-720Em.png]

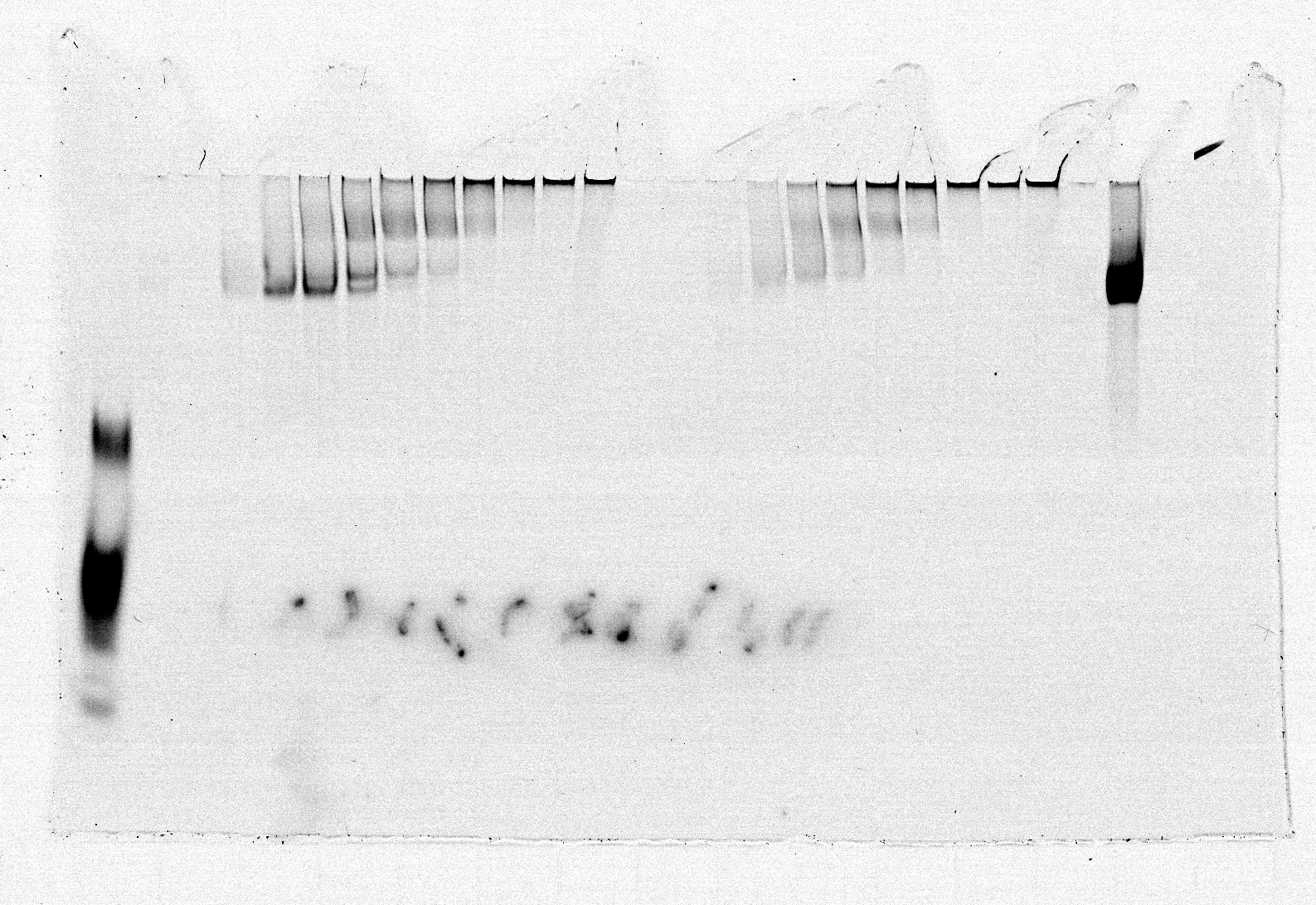

Supplement: Figure 3—source data 2. [file elife-103486-fig3-data2.zip › Fig3_source_data_2/0001777_01_488Ex-530Em.png]

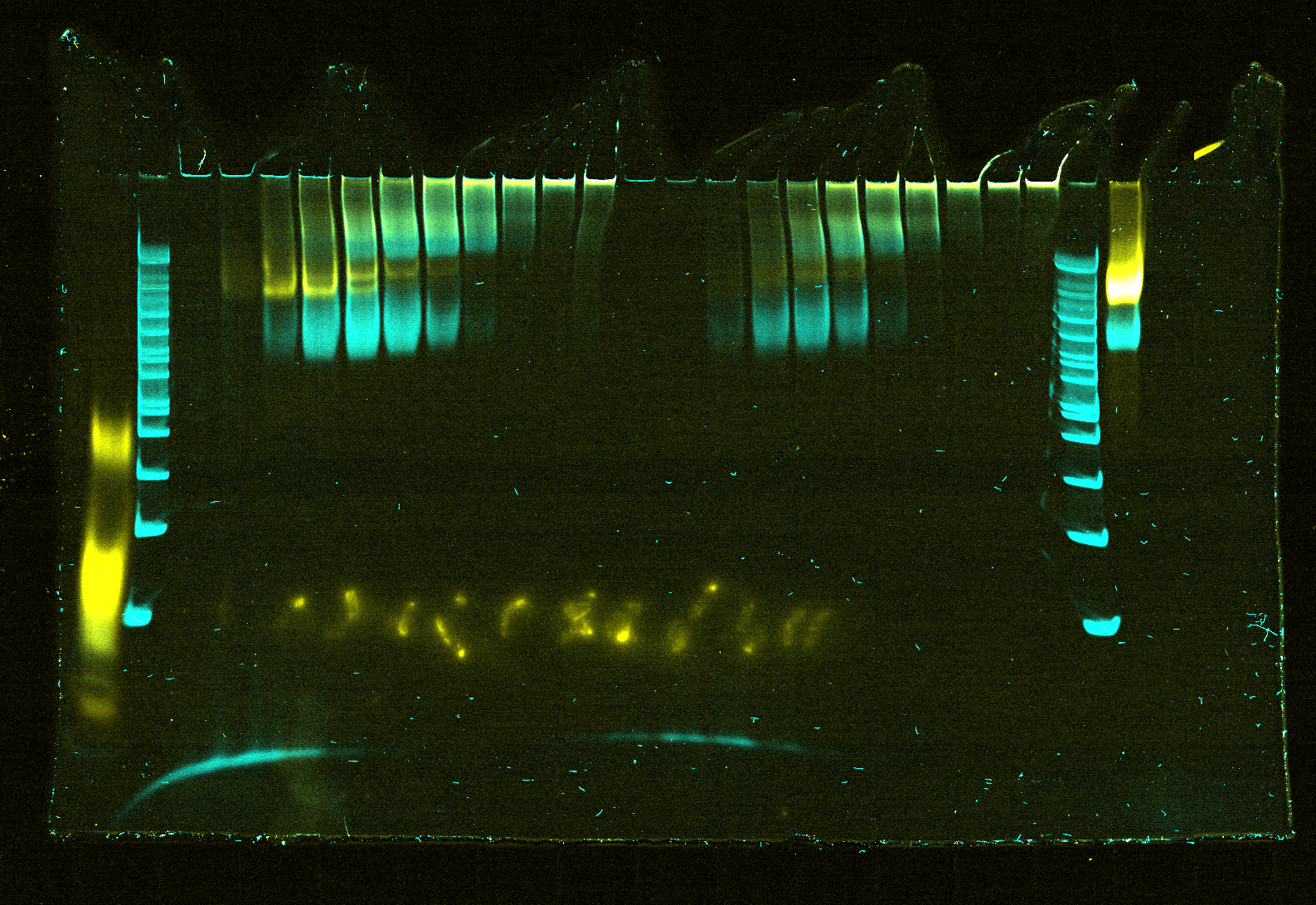

Supplement: Figure 3—source data 2. [file elife-103486-fig3-data2.zip › Fig3_source_data_2/Composite_GFP01_DNA5_CY.png]

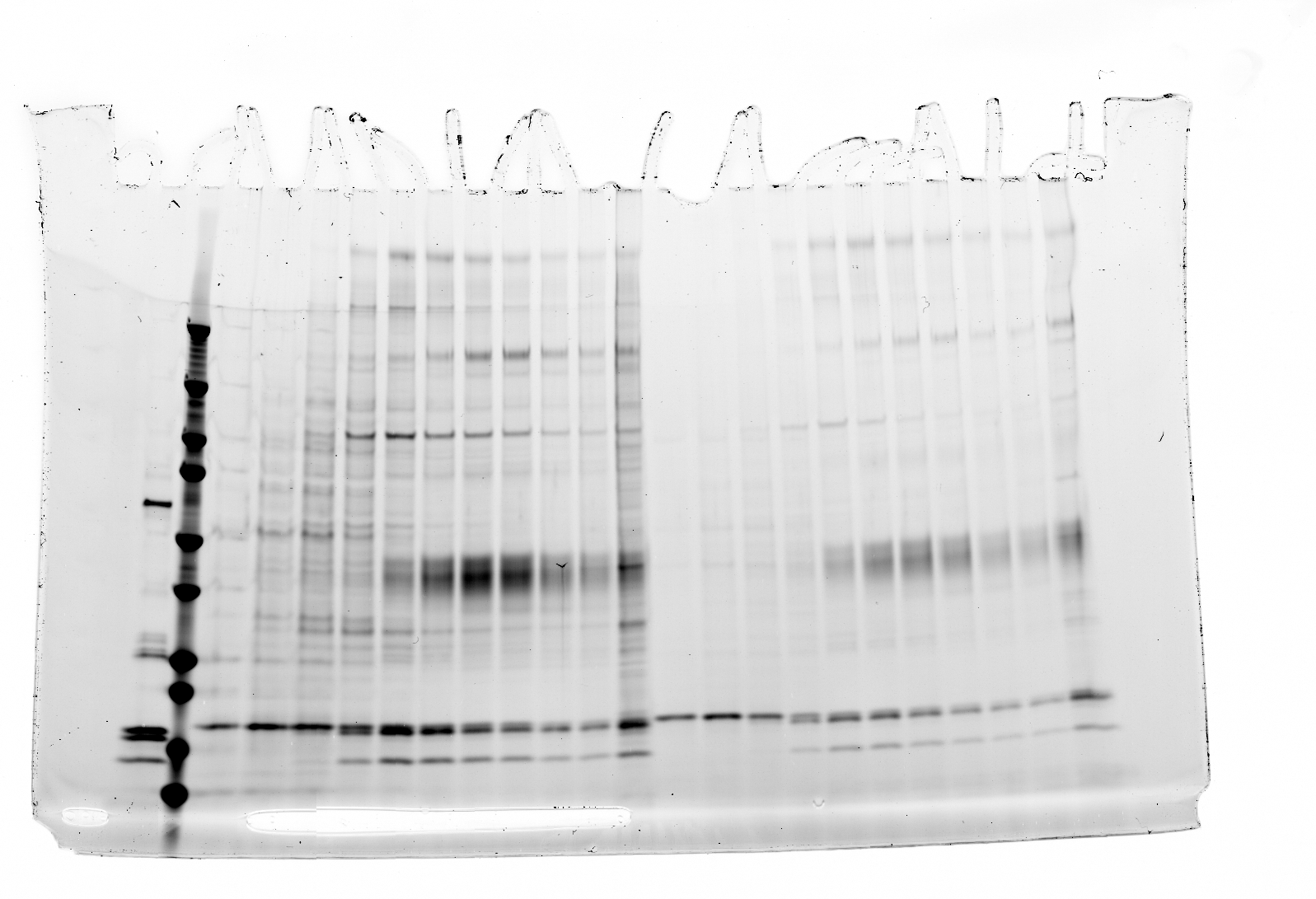

Supplement: Figure 3—source data 2. [file elife-103486-fig3-data2.zip › Fig3_source_data_2/0001786_01_685Ex-720Em.png]

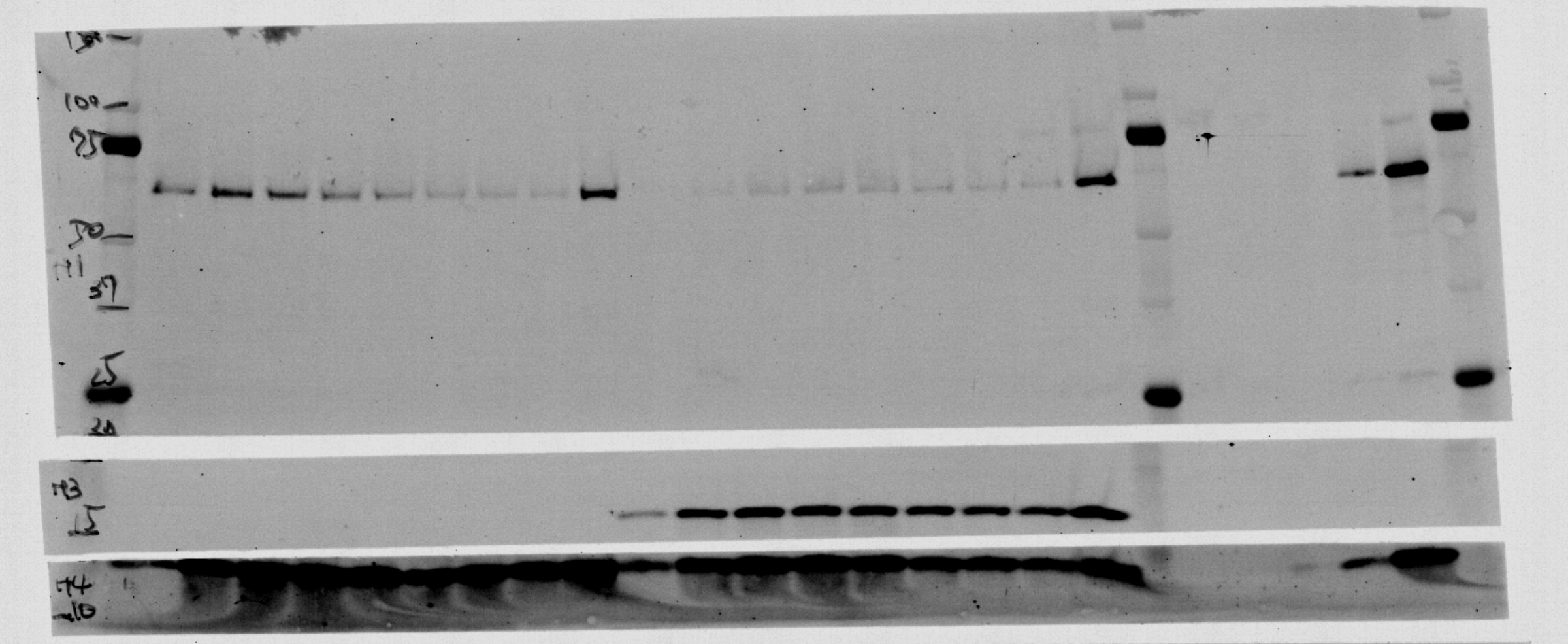

Supplement: Figure 3—source data 2. [file elife-103486-fig3-data2.zip › Fig3_source_data_2/0001797-002.png]

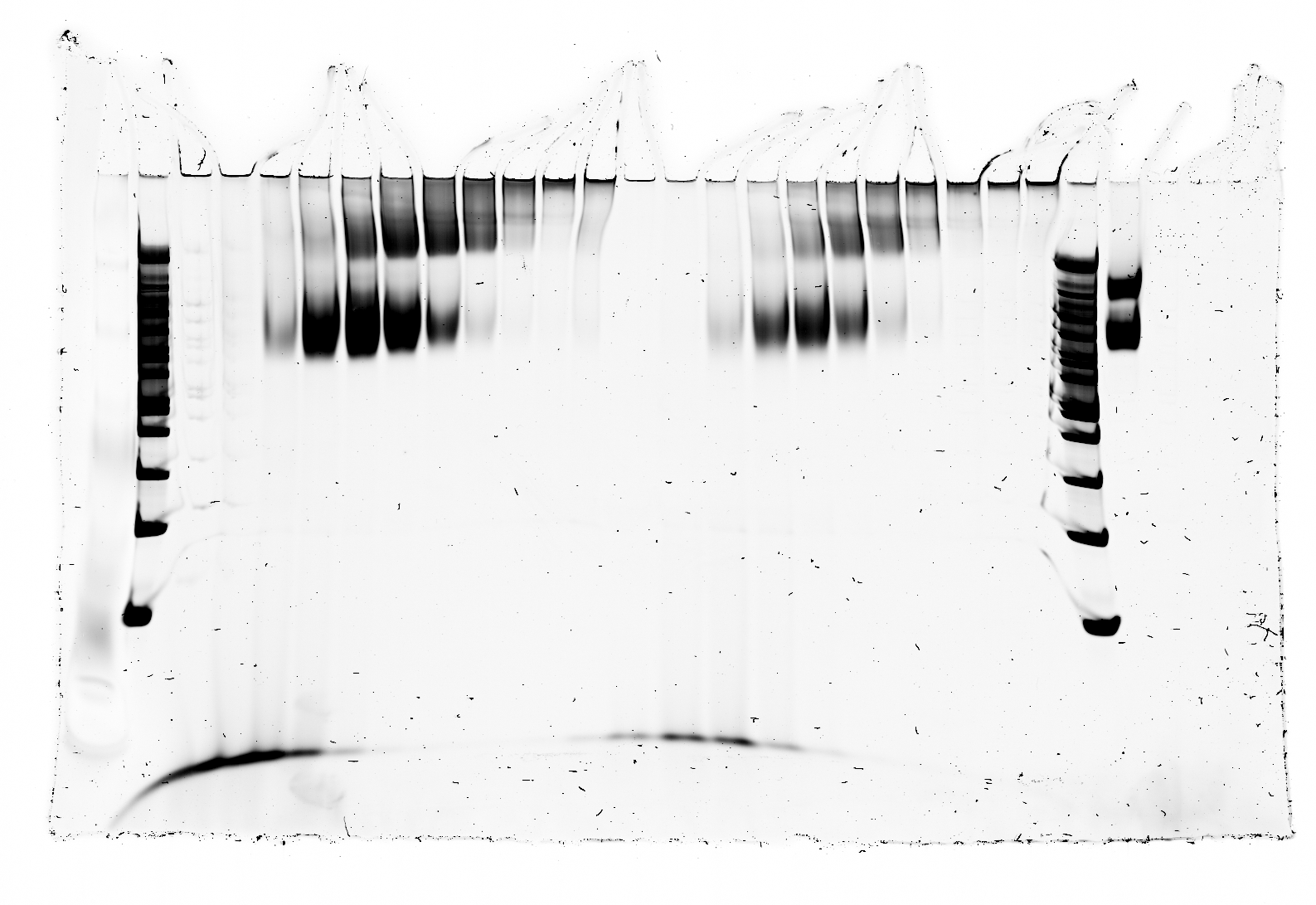

Supplement: Figure 3—source data 2. [file elife-103486-fig3-data2.zip › Fig3_source_data_2/0001777_01_685Ex-720Em.png]

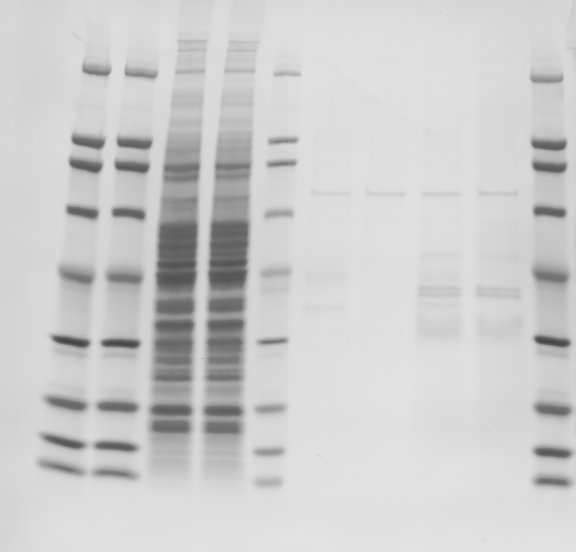

Supplement: Figure 3—figure supplement 1—source data 2. [file elife-103486-fig3-figsupp1-data2.zip › Fig3_Supple1_source_data_2/0003064_01_525Epi_raw1.jpg]

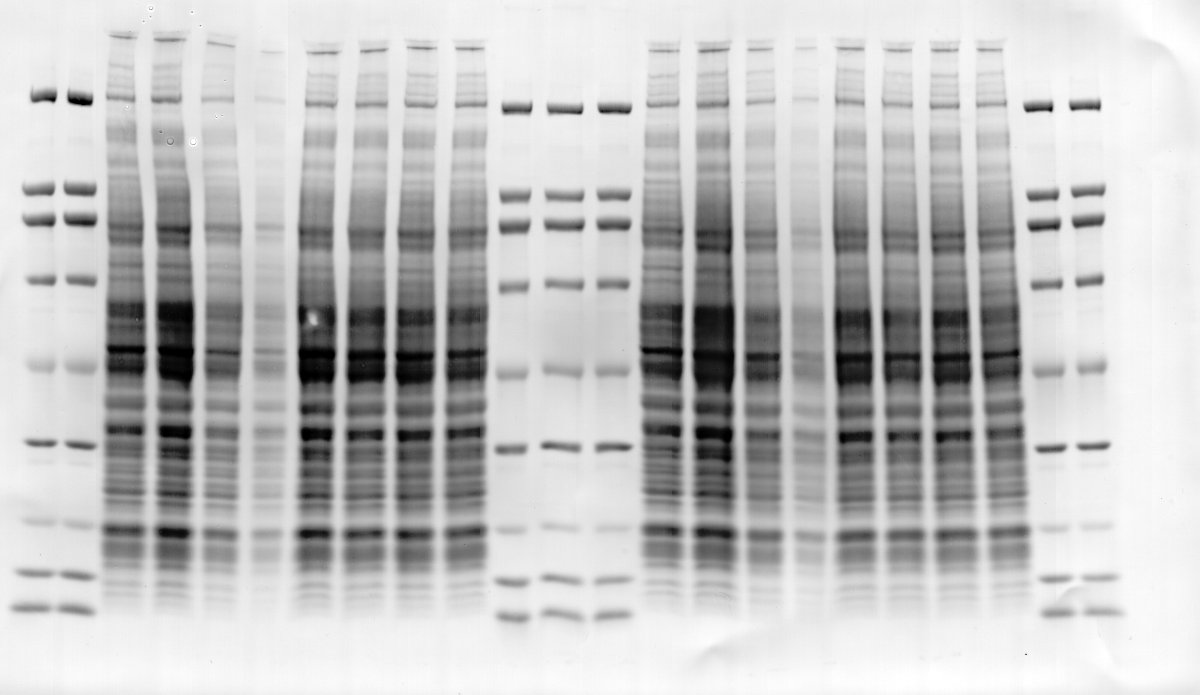

Supplement: Figure 3—figure supplement 1—source data 2. [file elife-103486-fig3-figsupp1-data2.zip › Fig3_Supple1_source_data_2/0002565_01_525Epi_raw1.jpg]

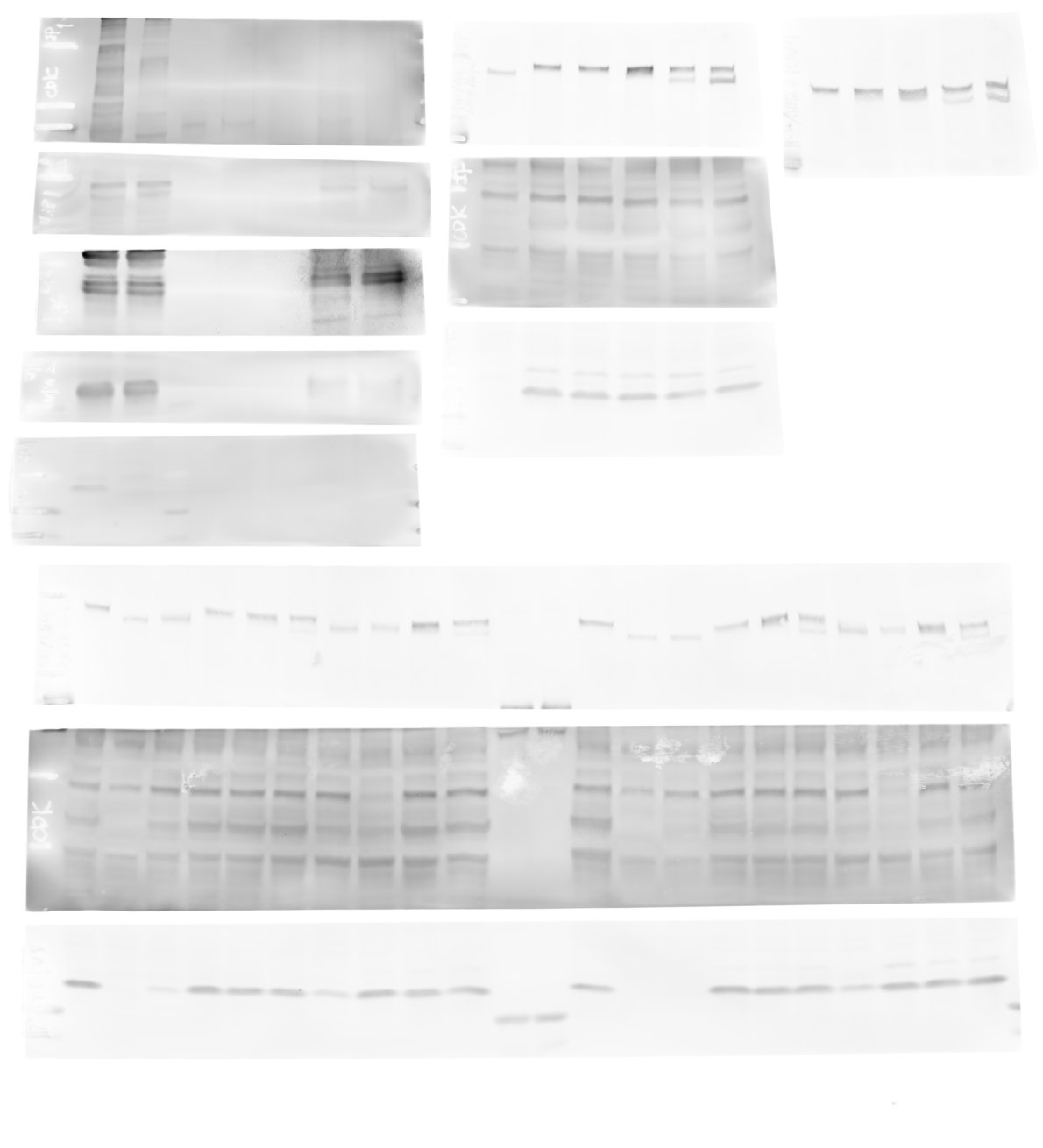

Supplement: Figure 3—figure supplement 1—source data 2. [file elife-103486-fig3-figsupp1-data2.zip › Fig3_Supple1_source_data_2/0003065_01_785Ex-820Em_raw3.jpg]

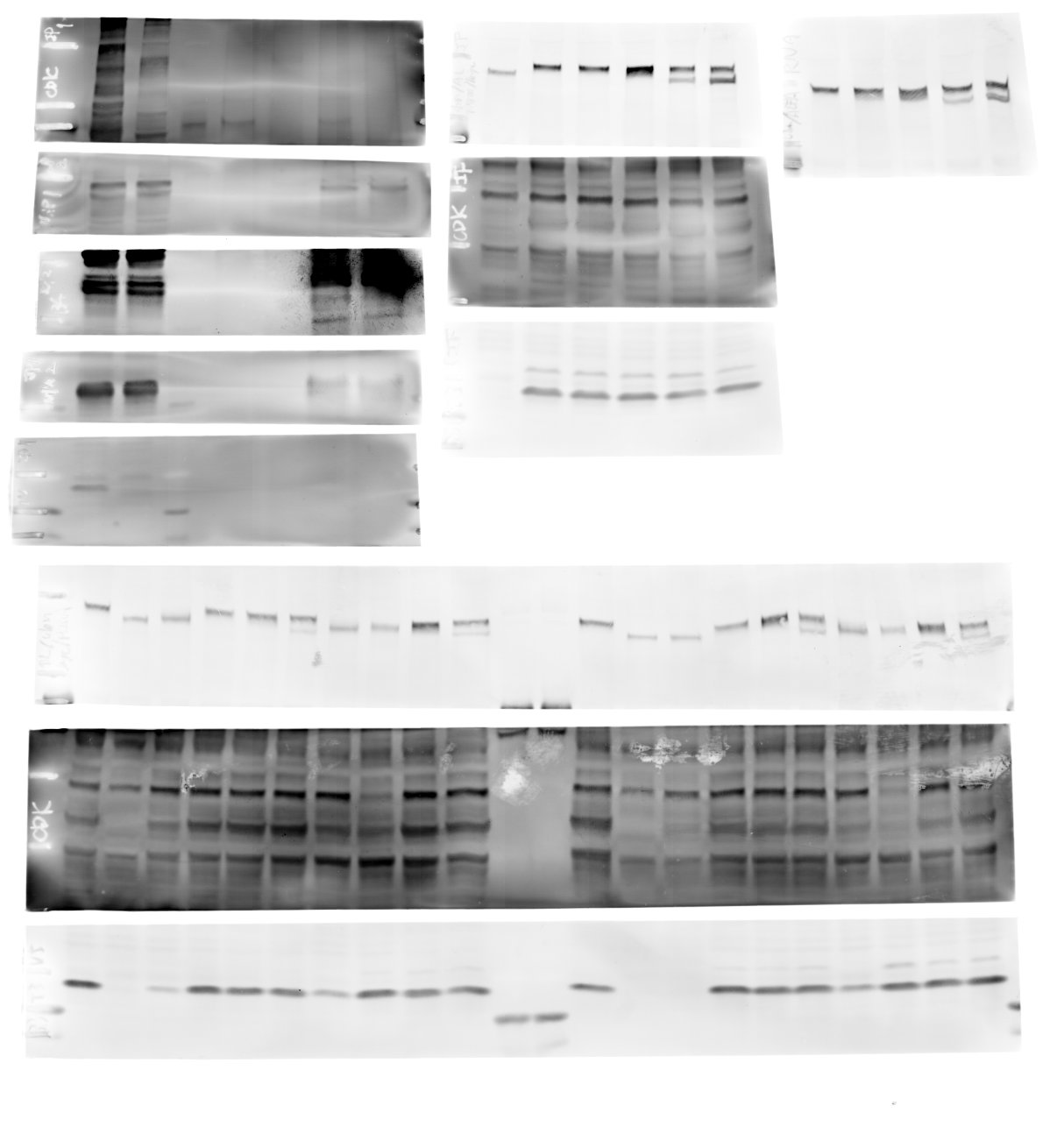

Supplement: Figure 3—figure supplement 1—source data 2. [file elife-103486-fig3-figsupp1-data2.zip › Fig3_Supple1_source_data_2/0003065_01_785Ex-820Em_raw3_1.jpg]

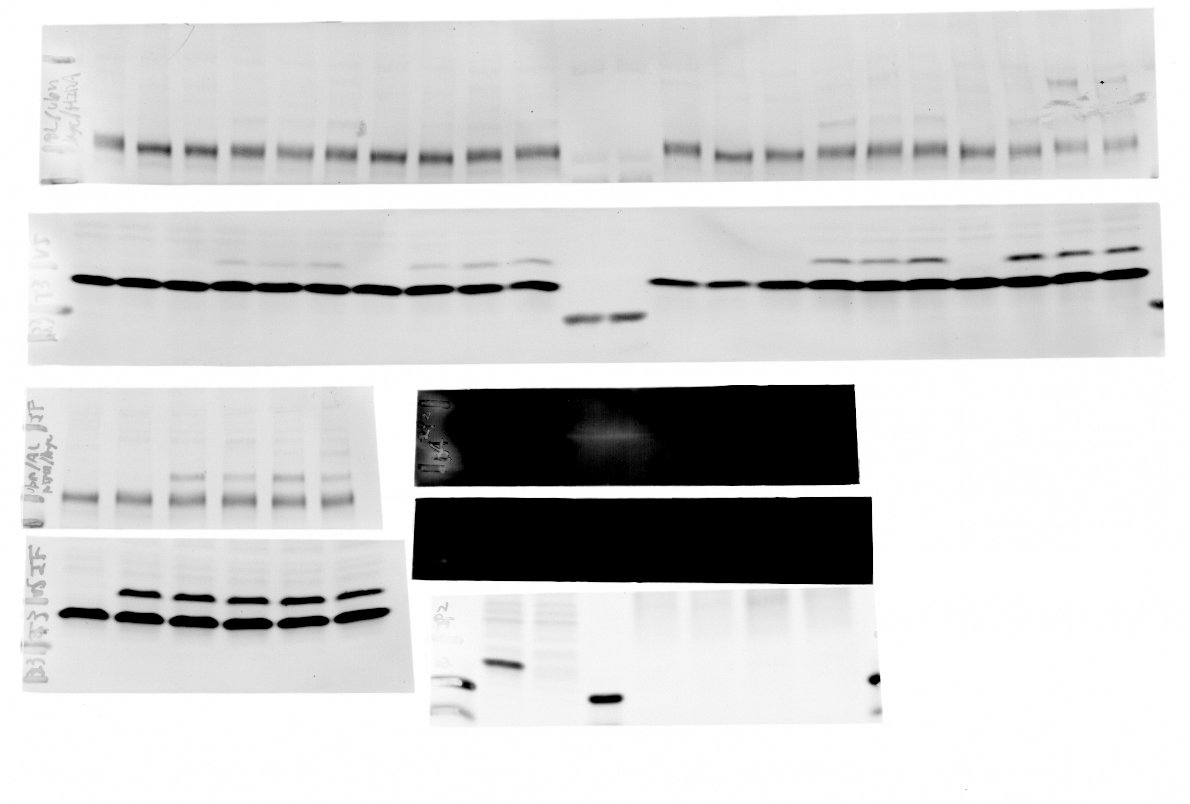

Supplement: Figure 3—figure supplement 1—source data 2. [file elife-103486-fig3-figsupp1-data2.zip › Fig3_Supple1_source_data_2/0003073_01_785Ex-820Em_raw2_1.jpg]

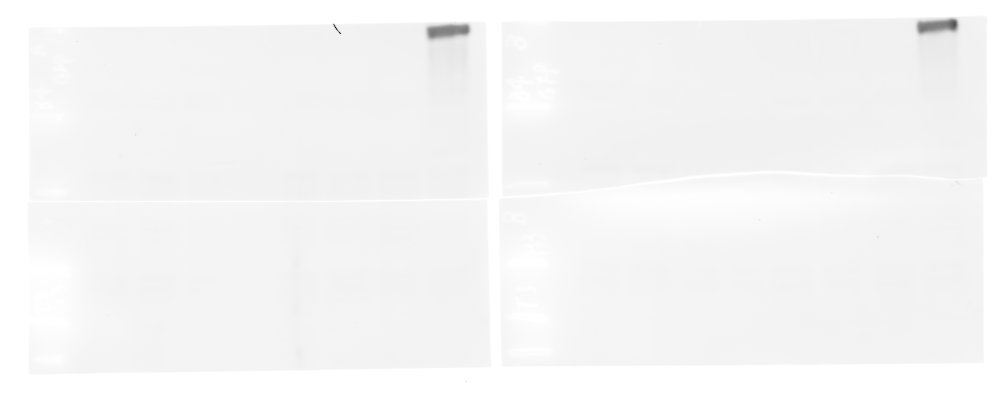

Supplement: Figure 3—figure supplement 1—source data 2. [file elife-103486-fig3-figsupp1-data2.zip › Fig3_Supple1_source_data_2/0002578_01_685Ex-720Em_raw3.jpg]

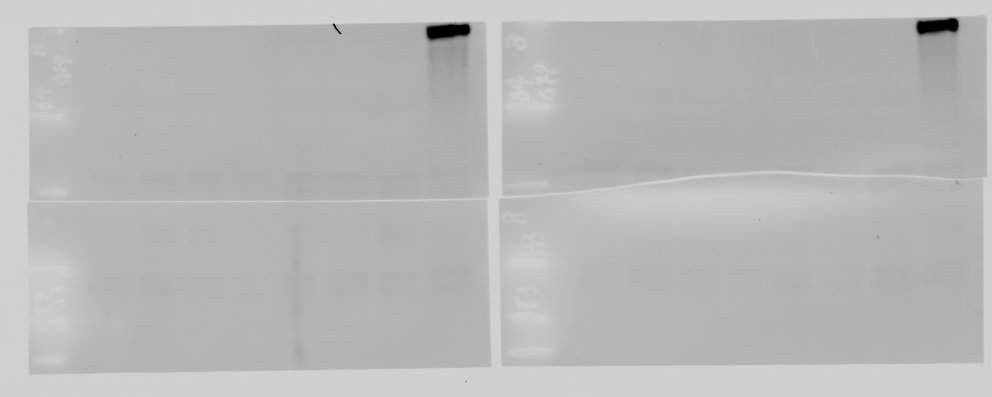

Supplement: Figure 3—figure supplement 1—source data 2. [file elife-103486-fig3-figsupp1-data2.zip › Fig3_Supple1_source_data_2/0002578_01_685Ex-720Em_raw3_bc.jpg]

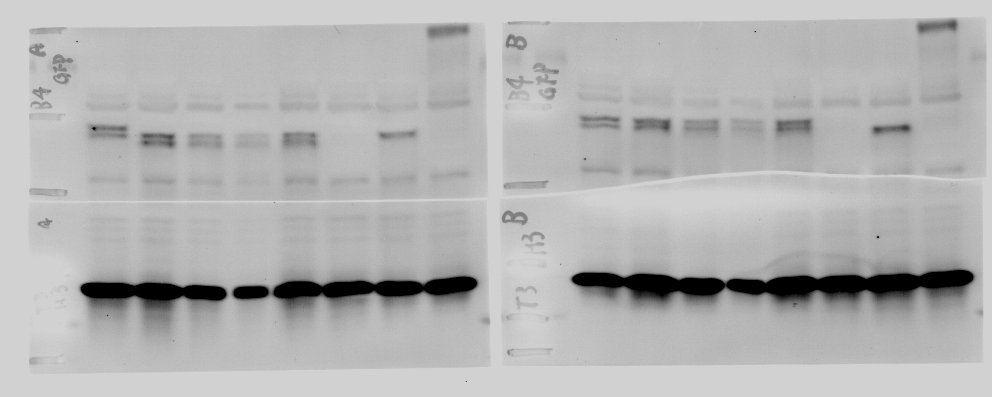

Supplement: Figure 3—figure supplement 1—source data 2. [file elife-103486-fig3-figsupp1-data2.zip › Fig3_Supple1_source_data_2/0002578_01_785Ex-820Em_raw2_bc.jpg]

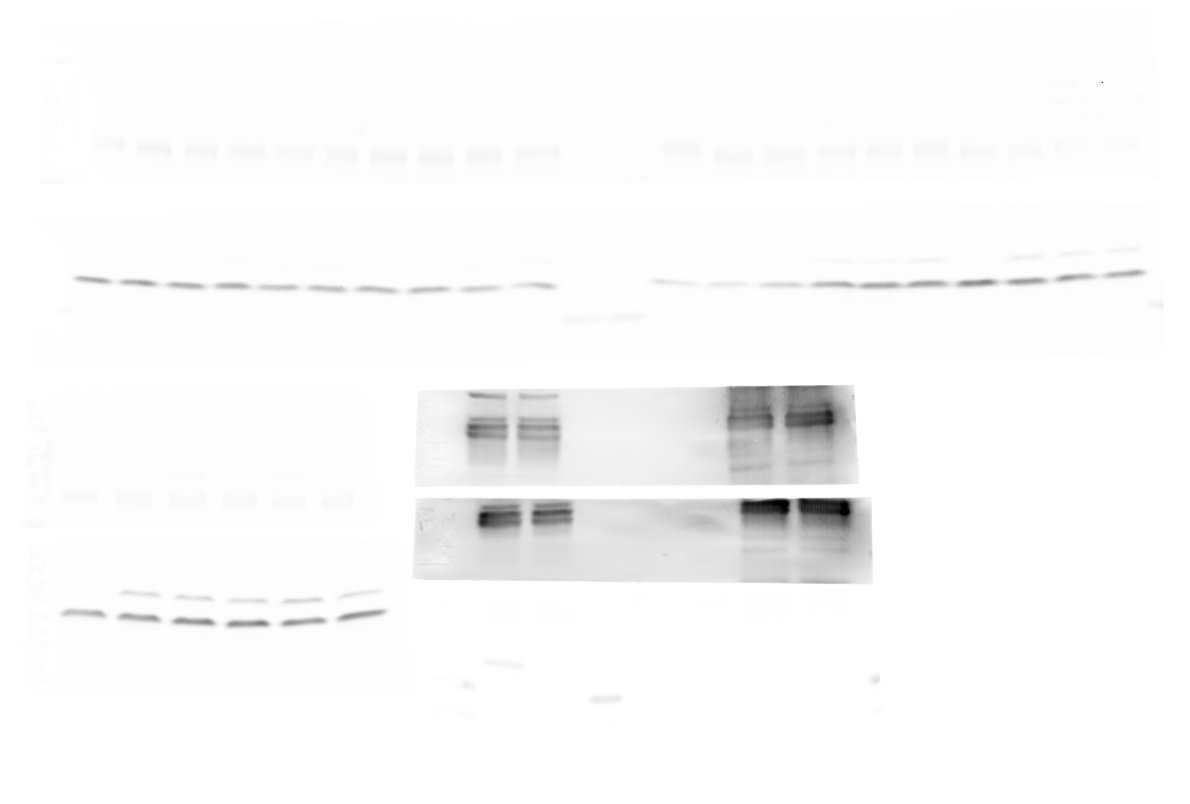

Supplement: Figure 3—figure supplement 1—source data 2. [file elife-103486-fig3-figsupp1-data2.zip › Fig3_Supple1_source_data_2/0003073_01_785Ex-820Em_raw2.jpg]

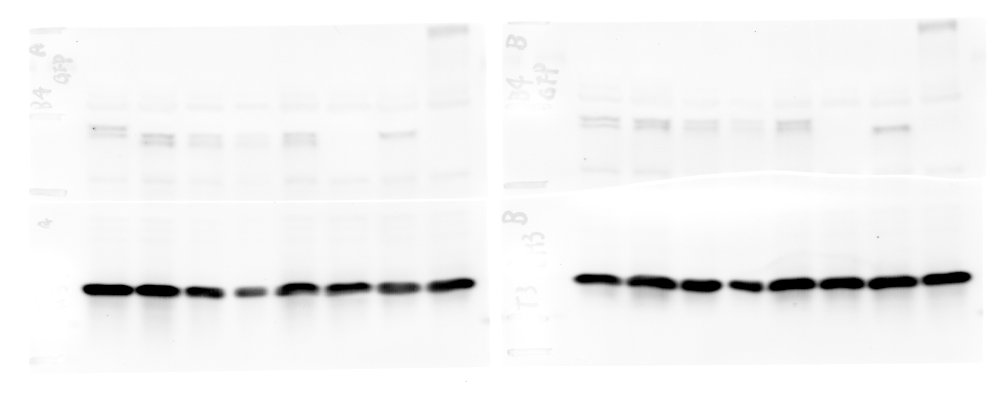

Supplement: Figure 3—figure supplement 1—source data 2. [file elife-103486-fig3-figsupp1-data2.zip › Fig3_Supple1_source_data_2/0002578_01_785Ex-820Em_raw2.jpg]

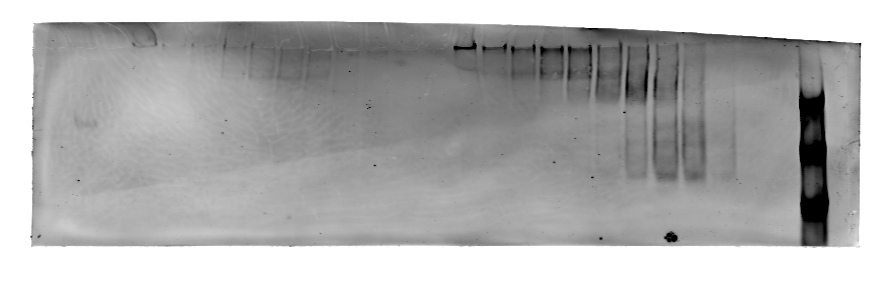

Supplement: Figure 3—figure supplement 2—source data 2. [file elife-103486-fig3-figsupp2-data2.zip › Fig3_Supple2_source_data_2/0013426_01_700.png]

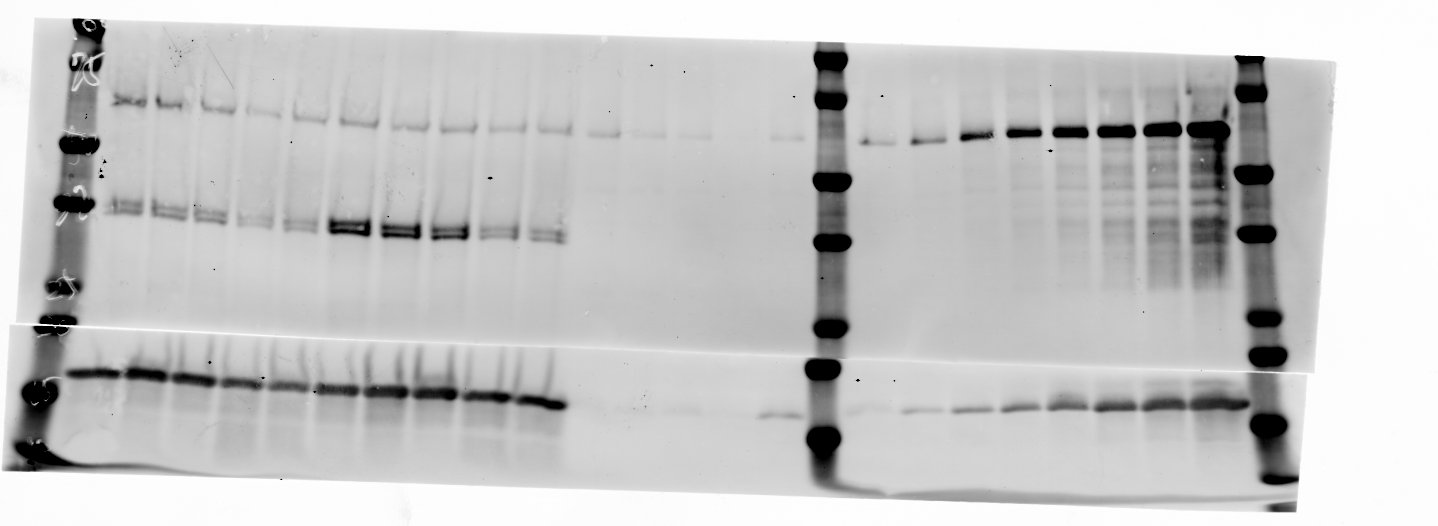

Supplement: Figure 3—figure supplement 2—source data 2. [file elife-103486-fig3-figsupp2-data2.zip › Fig3_Supple2_source_data_2/0001881_01_685Ex-720Em_0to10.jpg]

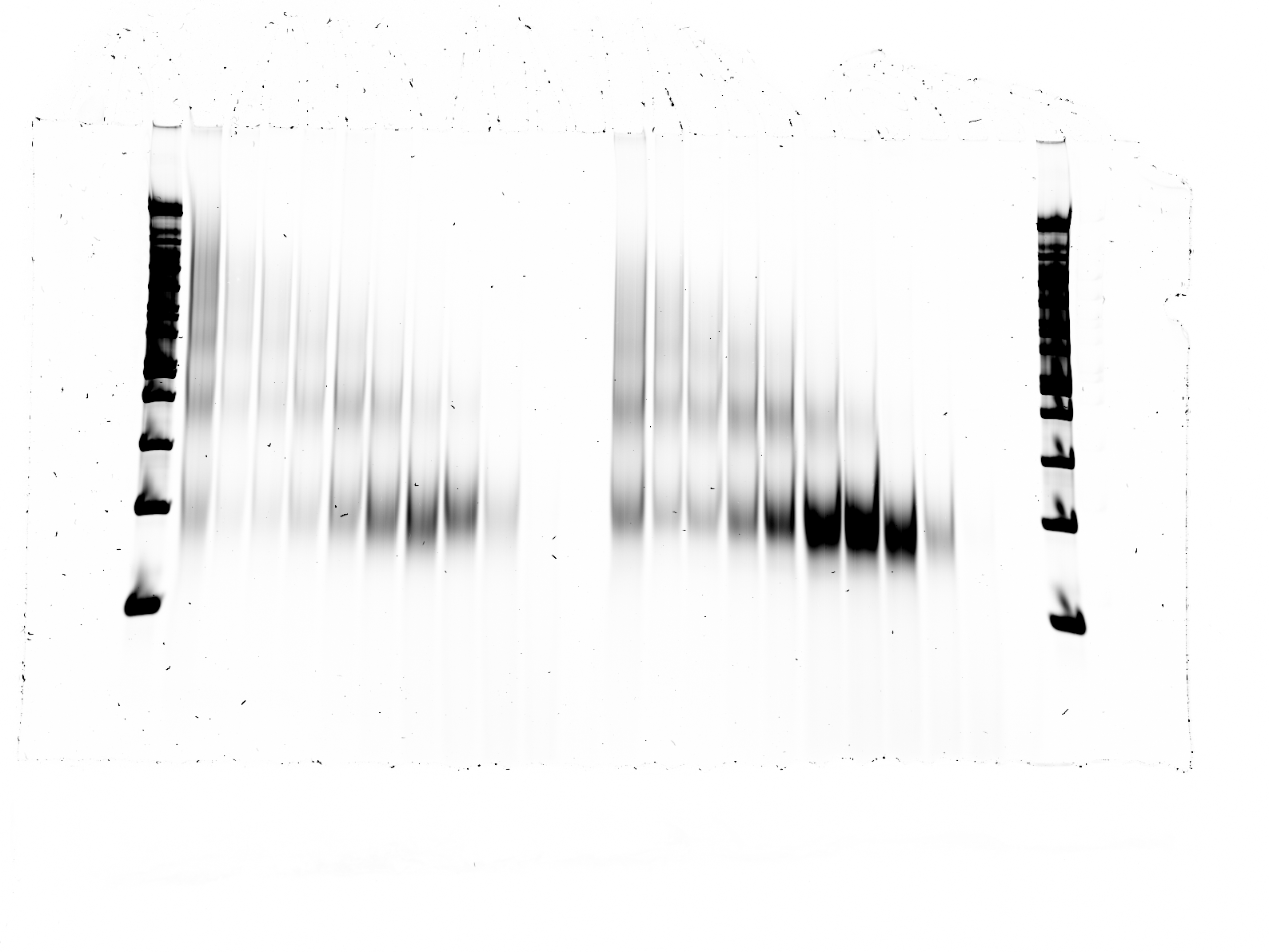

Supplement: Figure 3—figure supplement 2—source data 2. [file elife-103486-fig3-figsupp2-data2.zip › Fig3_Supple2_source_data_2/0001810_01_488Ex-530Em.png]

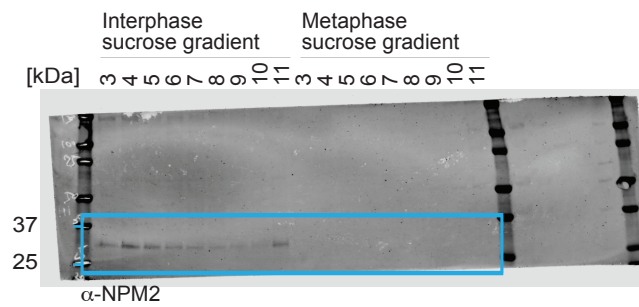

**Figure 4—source data 1.** Full images of gels and membranes shown in Figure3E

Supplement: Figure 4—source data 1. [file elife-103486-fig4-data1.pdf]
