# Supplementary material for: MagIC-Cryo-EM, structural determination on magnetic beads for scarce macromolecules in heterogeneous samples
Source: eLife. 2025 May 20;13:RP103486. doi: 10.7554/eLife.103486 (PMC12092007; doi:10.7554/eLife.103486)
Supplement: Figure 3—source data 1. — (A) Full gel images used in Figure 3C. (B) Full membrane image used in Figure 3D. (C) Full gel image used in Figure 3E. [file elife-103486-fig3-data1.pdf]

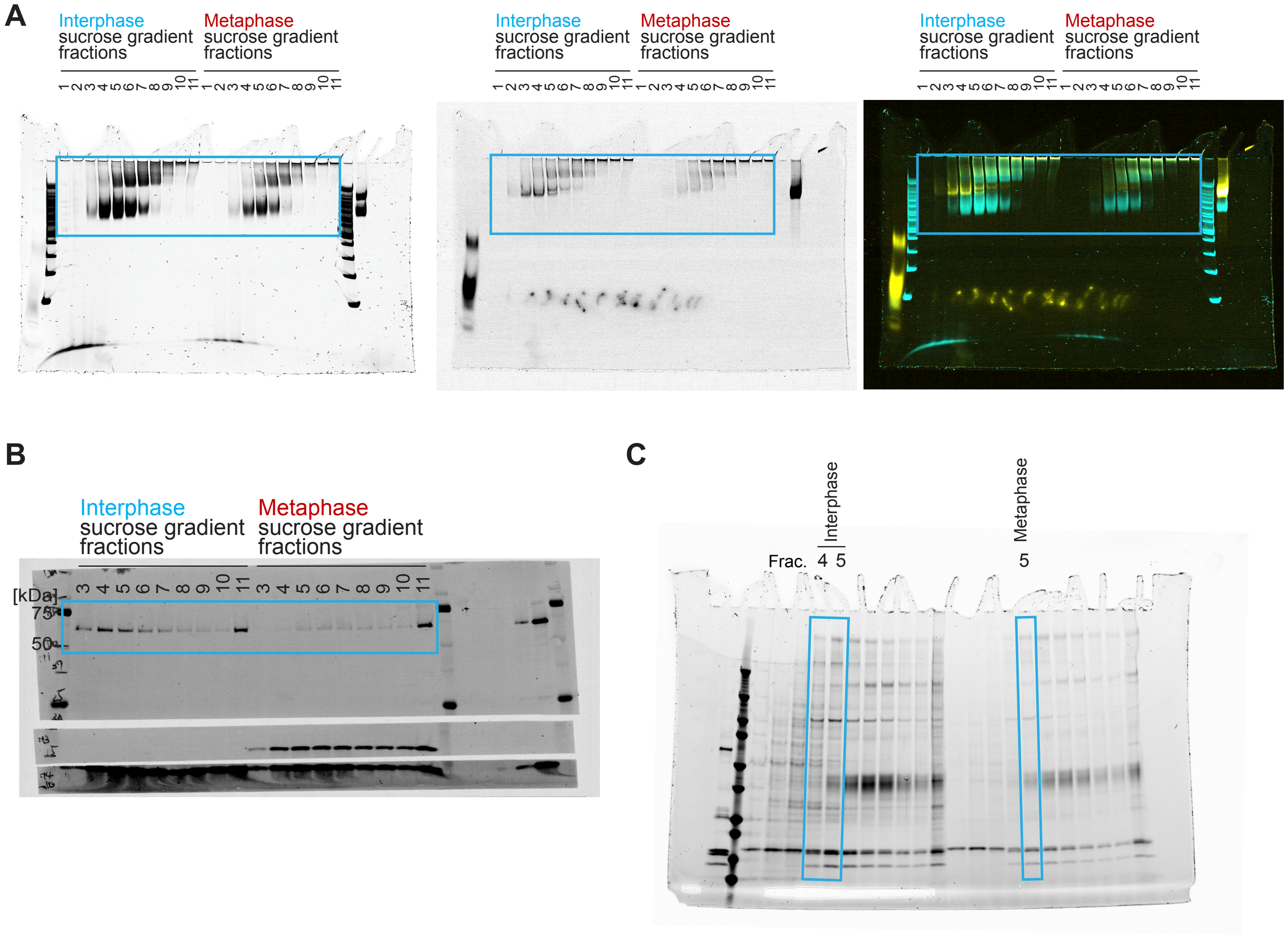


**Figure 3—source data 1.** Full images of gels and membranes shown in Figure3 **(A)** Full gel images used in Figure 3C. **(B)** Full membrane image used in Figure 3D. **(C)** Full gel image used in Figure 3E.
