# Supplementary material for: MagIC-Cryo-EM, structural determination on magnetic beads for scarce macromolecules in heterogeneous samples
Source: eLife. 2025 May 20;13:RP103486. doi: 10.7554/eLife.103486 (PMC12092007; doi:10.7554/eLife.103486)
Supplement: Figure 3—figure supplement 1—source data 1. — (A) Full gel images used in Figure 3—figure supplement 1A. (B) Full gel image used in Figure 3—figure supplement 1B. [file elife-103486-fig3-figsupp1-data1.pdf]

A

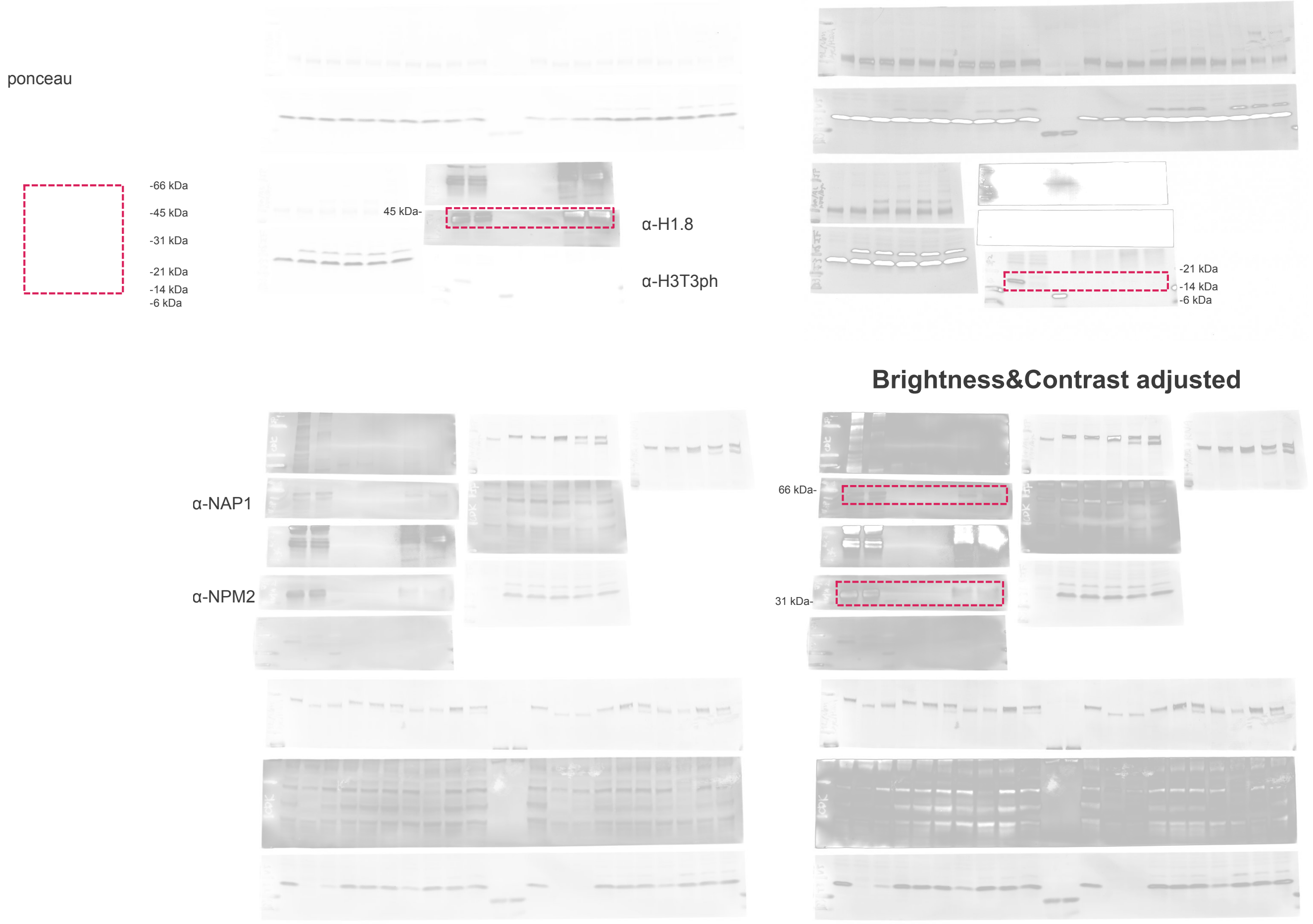

B

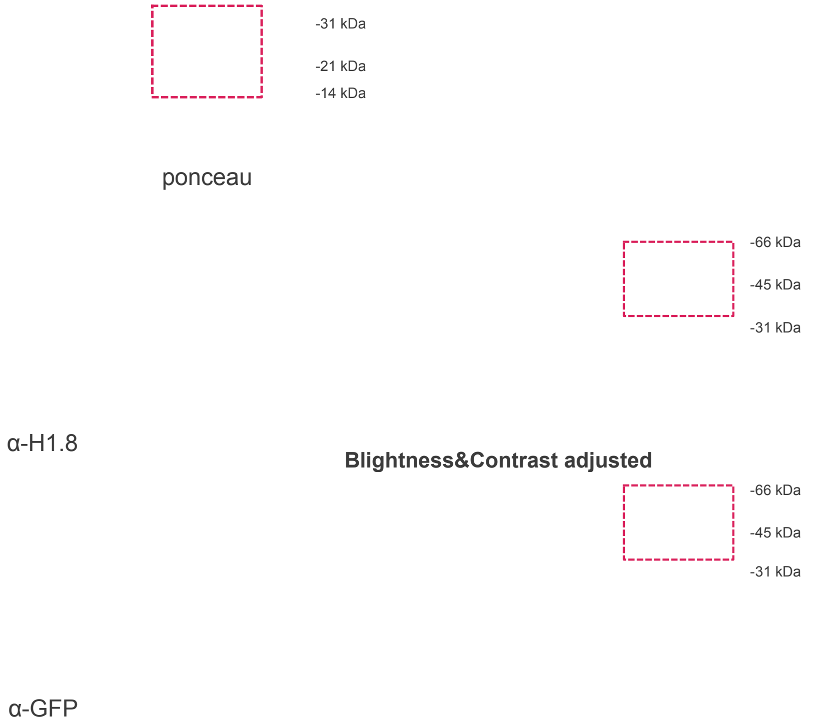

Figure 3—figure supplement 1—source data 1. Full images of gels and membranes shown in Figure3—figure supplement 2 **(A)** Full gel images used in Figure3—figure supplement 1A. **(B)** Full gel image used in Figure3—figure supplement 1B.
