# Supplementary material for: MagIC-Cryo-EM, structural determination on magnetic beads for scarce macromolecules in heterogeneous samples
Source: eLife. 2025 May 20;13:RP103486. doi: 10.7554/eLife.103486 (PMC12092007; doi:10.7554/eLife.103486)
Supplement: Figure 3—figure supplement 2—source data 1. — (A) Full gel images used in Figure 3—figure supplement 2A. (B) Full gel image used in Figure 3—figure supplement 2B. (C) Full membrane image used in Figure 3—figure supplement 2C. (D) Full membrane images used in Figure 3—figure supplement 2D. [file elife-103486-fig3-figsupp2-data1.pdf]

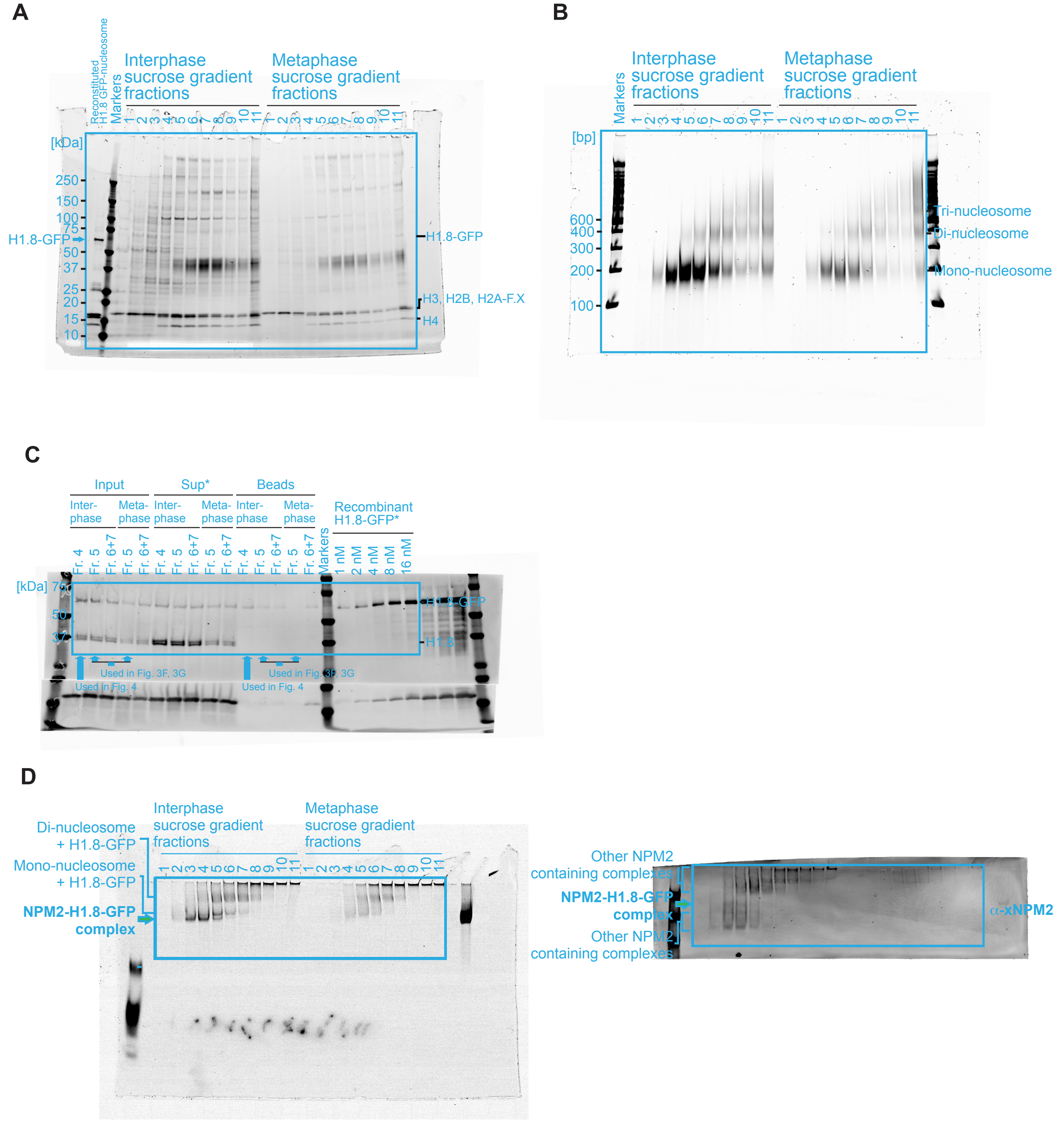

Figure 3—figure supplement 2—source data 1. Full images of gels and membranes shown in Figure3—figure supplement 2 **(A)** Full gel images used in Figure3—figure supplement 2A. **(B)** Full gel image used in Figure3—figure supplement 2B. **(C)** Full membrane image used in Figure3—figure supplement 2C. **(D)** Full membrane images used in Figure3—figure supplement 2D.
